# Sample Preparation of Posaconazole Oral Suspensions for Identification of the Crystal Form of the Active Pharmaceutical Ingredient

**DOI:** 10.3390/molecules25246032

**Published:** 2020-12-19

**Authors:** Michail Lykouras, Stefani Fertaki, Malvina Orkoula, Christos Kontoyannis

**Affiliations:** 1 Department of Pharmacy, University of Patras, GR-26504 Rio Achaias, Greece; michalislyk@gmail.com (M.L.); sfertaki@gmail.com (S.F.); malbie@upatras.gr (M.O.); 2 Institute of Chemical Engineering Sciences, Foundation of Research and Technology-Hellas (ICE-HT/FORTH), GR-26504 Platani Achaias, Greece

**Keywords:** posaconazole, oral suspension, polymorphism, form-S, sample preparation, centrifugation, XRPD

## Abstract

Determination of the polymorphic form of an active pharmaceutical ingredient (API) in a suspension could be really challenging because of the water phase and the low concentration of the API in this formulation. Posaconazole is an antifungal drug available also as an oral suspension. The aim of this study was to develop a sample-preparation method for polymorphic identification of the dispersed API by increasing the concentration of the API but with no compromise of polymorph stability. For this purpose, filtration, drying and centrifugation were tested for separating the API from the suspending medium. Centrifugation was selected because it succeeded in separating Posaconazole API with no polymorph transformation during the process. During this study, it was found that Posaconazole in oral suspensions is Form-S. However, when slower scanning rates were used for acquiring an XRPD pattern with better signal/noise ratio, Posaconazole was converted to Form I due to water loss. In order to protect the sample from conversion, different approaches were tested to secure an airtight sample including a commercially available XRPD sample holder with a dome-like transparent cap, standard polymethylmethacrylate (PMMA) sample holders covered with Mylar film, transparent pressure-sensitive tape and a transparent food membrane. Only usage of the transparent food membrane was found to protect the API from conversion for a period of at least two weeks and resulted in a Posaconazole Form-S XRPD pattern with no artificial peaks.

## 1. Introduction

Solid Active Pharmaceutical Ingredients (APIs) might exist in various crystal forms with different physical properties, although their chemical properties are identical. The multiple crystal forms are known as polymorphs, and the phenomenon is called polymorphism [1]. The crystal forms created by the combination of API molecules and solvent molecules in different stoichiometric proportions are known as solvates. When the solvent is water, the crystal form is a hydrate. Pseudopolymorphism is the term that describes the behaviour of solvates and hydrates [2]. Another form of solid APIs is the amorphous solid lacking long-range order or well-defined conformation of API molecules [3].

The phenomenon of polymorphism is of high importance for the pharmaceutical industry because the phase transition of an API could lead to undesirable properties affecting the stability of the final product, influencing the dissolution properties and the bioavailability of the API, and hence generating different pharmacological action [4]. Therefore, identification of the polymorphic or pseudopolymorphic form of the API in the final pharmaceutical product is considered critical for the development of the product.

Chromatographic techniques could not be applied for the qualitative determination of the crystal form of an API because different polymorphs cannot be identified by these methods. Among the analytical techniques used for polymorphic identification of a solid API, methods at the molecular level, particulate level and bulk level are included. The first class of methods involves spectroscopic methods, like Infrared Spectroscopy (IR), Raman Spectroscopy and solid-state Nuclear Magnetic Resonance (ss-NMR). The second category involves X-ray Powder Diffraction (XRPD); Light Microscopy; Scanning Electron Microscopy (SEM); and thermoanalytical methods, such as Differential Scanning Calorimetry (DSC), Thermogravimetric Analysis (TGA) and Dynamic Vapour Sorption (DVS). The last class involves the method of Karl Fisher Titration and the Brunauer, Emmett and Teller (BET) method [1].

Oral suspensions are pharmaceutical formulations widely preferred for paediatric or geriatric administration and are also characterized as dispersions because the solid API and possibly some excipients are dispersed as solid particles in a liquid vehicle or suspending medium [5]. Due to the liquid suspending medium, which is frequently water, the APIs in oral suspensions are prone to polymorphic transition causing physical instability of the suspension. This lack of physical stability can be expressed as crystal growth or caking, i.e., the loss of a suspension′s ability to be resuspended uniformly [6]. Therefore, identification of the API′s polymorph in an oral suspension is very important so that the stability of that pharmaceutical formulation is ensured.

However, determination of the polymorphic form of the API in an oral suspension is often challenging. The liquid phase of oral suspensions affecting the signal/noise ratio, the low concentration of the API in those pharmaceutical formulations and the lack of homogeneity of the samples are frequently the basic obstacles in the analysis methods used for their polymorphic characterization [7]. These challenges in the polymorphic characterization of the API in pharmaceutical oral suspensions in combination with the lack of suitable methods in the literature for overcoming these difficulties render the development of a novel sample-preparation method to be of high importance. The novel method to be developed should be capable of eliminating the interference of water contained in pharmaceutical oral suspensions without causing phase conversion of the API and at the same time differentiating the possible different polymorphs of the API in those formulations successfully.

For this purpose, Posaconazole oral suspensions were used. Posaconazole is a broad-spectrum member of the second generation antifungal triazole drugs and is used against invasive fungal infections [8,9]. It is commercially available as an oral suspension (40 mg/mL), as a concentrate for solution for intravenous infusion (300 mg) and as gastro-resistant tablets (100 mg) [10]. Although 12 different forms of Posaconazole have been already described [11,12,13,14,15,16,17,18], Posaconazole Form I is used for the production of Posaconazole oral suspension [19].

The quite low concentration of Posaconazole in oral suspensions (40 mg/mL) and the nature of the suspensions are obstacles in identification of the crystal form of the API in these formulations. Therefore, the aim of this study was to develop a sample-preparation method to artificially increase the concentration of the API in the suspension without causing a polymorphic conversion and to subsequently determine the crystal form of Posaconazole in the concentrated sample.

## 2. Results

### 2.1. Posaconazole Oral Suspension Analysis via XRPD

**Figure 1:**
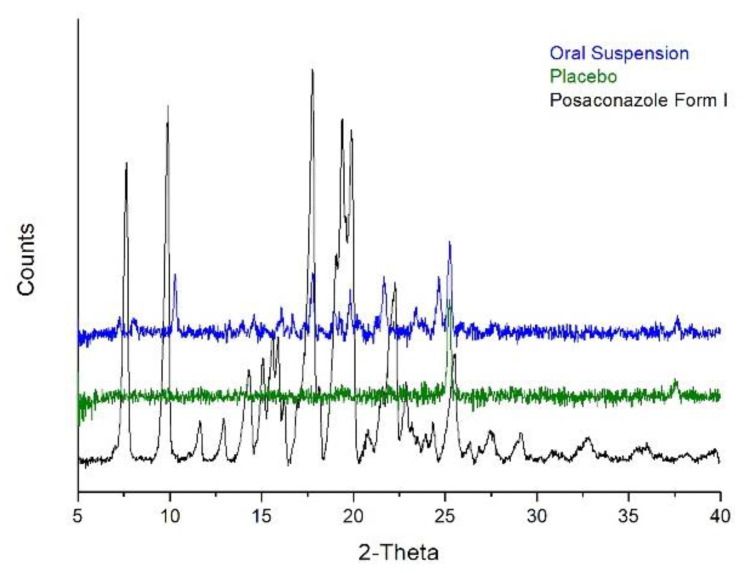
The XRPD patterns of Posaconazole oral suspension (**blue line**), Posaconazole oral suspension′s placebo (**green line**) and Posaconazole Form I (**black line**).

XRPD, Raman spectroscopy and Attenuated Total Reflectance Fourier-Transform Infrared (ATR/FT-IR) spectroscopy were tested for identification of the Posaconazole crystal form in Posaconazole oral suspension. However, the API could be barely detected via ATR/FT-IR spectroscopy in the as-received suspension and both techniques could not be used for differentiating among the different polymorphs of Posaconazole due to minor spectral differences. Hence, XRPD was selected for analysis of the Posaconazole oral suspension. The XRPD patterns of Posaconazole Form I, Posaconazole oral suspension′s placebo and Posaconazole oral suspension were recorded and compared to each other (Figure 1). Although Posaconazole Form I has been used to produce Posaconazole oral suspensions [19], the XRPD pattern of the oral suspensions revealed that these formulations were a mixture of Posaconazole oral suspension′s placebo and another polymorph or pseudopolymorph, or a combination of different forms of Posaconazole. The liquid phase of the oral suspension and the low concentration of the API in the suspension (40 mg/mL) resulted in a low signal/noise ratio in the XRPD pattern of Posaconazole oral suspension. These issues hindered identification of the crystal form of the API in the oral suspension, and it was not possible to determine whether the API in the oral suspension was found in one or more crystal forms at the same time.

### 2.2. Development of the Sample-Preparation Method for Increasing Posaconazole′s Concentration in Oral Suspensions

In order to overcome the problem of low signal/noise ratio, three different methods for increasing Posaconazole′s concentration in the sample were tested. Filtering, drying or centrifuging Posaconazole oral suspensions were suggested so that the suspending medium would be removed and the separated precipitate would contain Posaconazole API and the solid excipients of the oral suspensions. Filtration under vacuum failed to remove the suspending medium probably due to the presence of xanthan gum, which is used as a suspending agent of the API, which clogged the pores of the filters.

**Figure 2:**
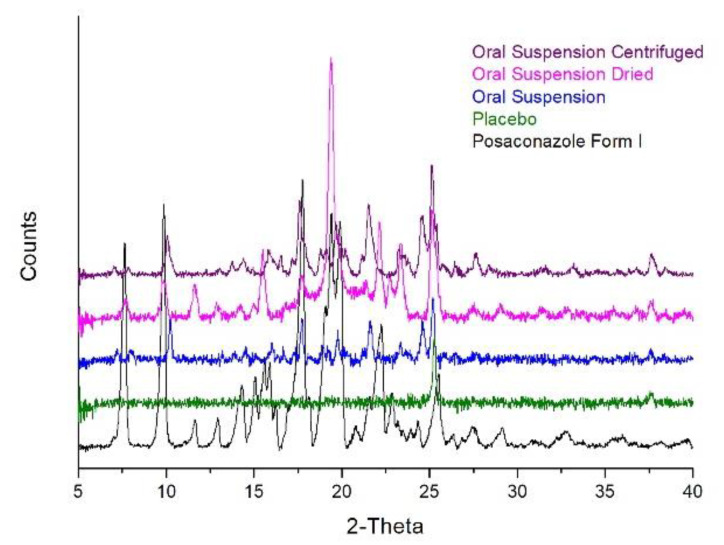
Development of the sample-preparation method for increasing Posaconazole′s concentration in oral suspensions: XRPD patterns of Posaconazole oral suspension′s precipitate after drying (**magenta line**) and after centrifugation (**purple line**), Posaconazole oral suspension (**blue line**), Posaconazole oral suspension′s placebo (**green line**) and Posaconazole Form I (black line).

The method of drying succeeded in removing the liquid phase of the suspension and in increasing Posaconazole′s concentration in the solid residue. More specifically, the final concentration of Posaconazole was approximately 12.5% *w*/*w*, which was more than triple in comparison to the initial concentration. However, the recorded XRPD pattern of the concentrated sample did not resemble the initial XRPD pattern of the oral suspension and highly resembled the pattern of Posaconazole Form I (Figure 2). Consequently, this sample-preparation method failed in retaining the polymorphic form of Posaconazole as it was in the oral suspension.

The final method tested for increasing the concentration of the API in the oral suspension was centrifugation. The precipitate after centrifugation was separated, containing Posaconazole API and the undissolved excipients. The concentration of the API in the precipitate was determined at approximately 20% *w*/*w*, i.e., it was increased more than 5 times in comparison to Posaconazole′s concentration in the oral suspension. The XRPD pattern of the precipitate highly resembled the pattern of the oral suspension, and its signal/noise ratio was increased (Figure 2). The acquired XRPD pattern was compared to the XRPD patterns of all known Posaconazole Forms [11,12,13,14,15,16,17,18], and it was found that Posaconazole in the collected precipitate was Form-S.

### 2.3. Setting the Scanning Rate of the XRPD Analysis

**Figure 3:**
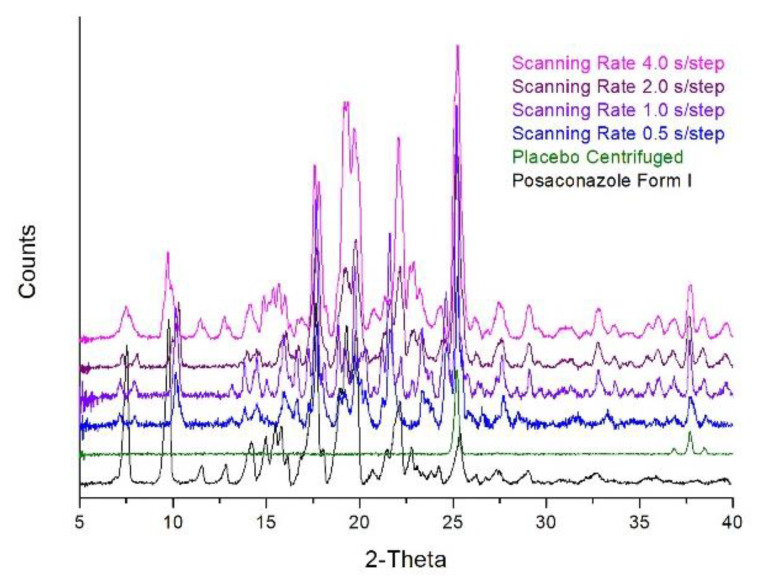
Setting the scanning rate of the XRPD analysis of the oral suspension′s precipitate: XRPD patterns of Posaconazole Form I (**black line**), precipitate of Posaconazole oral suspension′s placebo after centrifugation (**green line**) and precipitate of Posaconazole oral suspension after centrifugation recorded with scanning rate of 0.5 s/step (**blue line**), 1.0 s/step (**violet line**), 2.0 s/step (**purple line**) and 4.0 s/step (**magenta line**).

The effect of scanning rate on the signal/noise ratio of the XRPD pattern of the concentrated sample was investigated by applying scanning rates of 0.5 s/step, 1.0 s/step, 2.0 s/step and 4.0 s/step on fresh separated precipitates for each scanning rate. The XRD pattern which was acquired with a faster 0.5 s/step scanning rate (15 min scan) was used as the reference pattern against which all other XRD patterns were compared. When the 2-times slower scanning rate was applied (1.0 s/step, 30 min scan), it was observed that the quality of the pattern (signal/noise ratio) was improved; however, the pattern in the region 22–40 2-theta did not match the pattern recorded with the 0.5 s/step scanning speed. Although the pattern in the region 4–22 2-theta was identical to the sum of the centrifuged placebo and Posaconazole Form-S, in the region 22–40 2-theta, additional peaks attributed to Posaconazole Form I were also detected (Figure 3). Similar findings were also observed when a 2.0 s/step scanning rate (60 min scan) was applied. In the region 20–40 2-theta, additional peaks have been detected that could be attributed to Posaconazole Form I. As a result, the first half of the pattern highly resembled the sum of Posaconazole Form-S and the centrifuged placebo, while the second half matched the sum of Posaconazole Form I, Posaconazole Form-S and the centrifuged Placebo (Figure 3). When using the 8-times slower 4.0 s/step scanning rate (120 min scan), peaks of Posaconazole Form I could be detected across the XRD pattern (Figure 3). Therefore, it was concluded that, although the signal/noise ratio was improved, Posaconazole Form-S was susceptible to polymorphic conversion to Posaconazole Form I when slow scanning rates are used.

### 2.4. Stability of Posaconazole API in the Oral Suspension′s Precipitate

**Figure 4:**
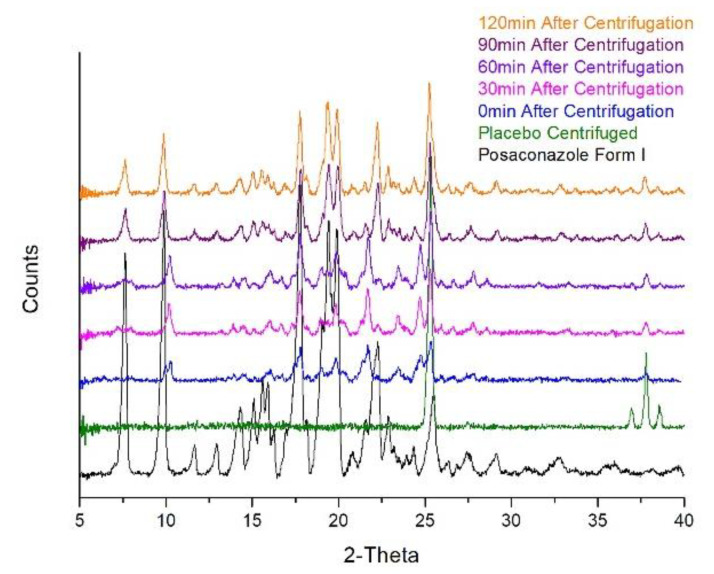
Stability of Posaconazole Form-S in the oral suspension′s precipitate: XRPD patterns of Posaconazole Form I (**black line**), precipitate of Posaconazole oral suspension′s placebo (**green line**) and precipitate of Posaconazole oral suspension immediately after centrifugation (**blue line**), 30 min (**magenta line**), 60 min (**violet line**), 90 min (**purple line**) and 120 min (**orange line**).

Because of the polymorphic conversion observed during XRPD analysis using a slower scanning rate, the stability of Posaconazole Form-S in the precipitate was studied at ambient temperature. This investigation led to the detection of a quick conversion to Posaconazole Form I. More specifically, immediately after centrifugation and for the next 30 min, the XRPD pattern of the precipitate was practically identical to the sum of Posaconazole Form-S and Posaconazole placebo. After 60 min since isolation of the precipitate by centrifugation, the conversion to Form I had already started and it was completed after 90 min. No other conversion was observed after this time point (Figure 4). These results were quite in accordance with the XRPD patterns recorded at different scanning rates. However, the XRPD pattern recorded with a 1.0 s/step scanning rate after the 22 2-theta region was a mixture of Posaconazole Form-S and Form I, although the duration of the analysis was only 30 min, while in stability tests, at ambient temperature, Posaconazole remained in Form-S for that period of time (Figure 4). An explanation to this could be the higher temperature in the X-ray diffractometer chamber during analysis and consequently the higher water loss from the precipitate leading to polymorphic conversion to Posaconazole Form I. Based on the above observation, it is highly possible that Posaconazole Form-S is likely to be a hydrate form.

### 2.5. Development of the Sample-Preparation Method for Delaying the Polymorphic Conversion

In order to prevent water loss from Posaconazole oral suspension′s precipitate and subsequent transformation of Form-S to Form I, the following protective materials were applied: a commercially available airtight XRPD sample holder with an X-ray transparent dome-like cap, Mylar film, transparent pressure-sensitive tape and a transparent food membrane. After the application of each protective material, the respective XRPD pattern was recorded and compared against each other and against the pattern of the uncovered centrifuged Posaconazole oral suspension.

**Figure 5:**
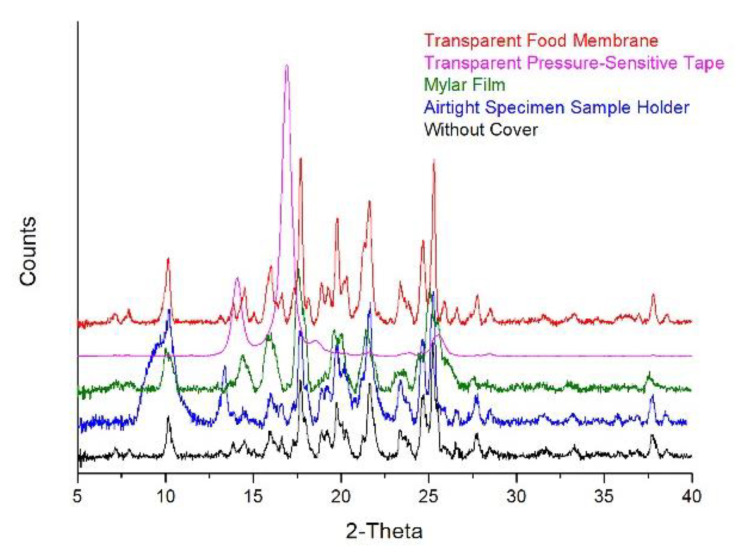
Sample-preparation methods for delaying polymorphic conversion of Posaconazole in the received precipitate after centrifuging Posaconazole oral suspension: XRPD patterns of Posaconazole precipitate without any cover (**black line**) and Posaconazole precipitates placed on an airtight specimen sample holder with dome-like X-ray transparent cap (**blue line**), on a PMMA sample holder covered with a Mylar film (**green line**), on a PMMA sample holder covered with transparent pressure-sensitive tape (**magenta line**) and on a PMMA sample holder enfolded with a transparent food membrane (**red line**).

The airtight specimen XRPD sample holder with the dome-like transparent cap, although ideal for environmentally sensitive materials, resulted in an XRPD pattern with an extra broad peak in the region 8.3–12.1 2-theta and an intense additional peak at 13.4 2-theta, which interfered with characteristic peaks of Posaconazole Form I and Form-S (Figure 5).

The Mylar film was used to cover the precipitate on a standard PMMA sample holder. The resulting XRPD pattern had a low signal/noise ratio, the peaks were rather broad and neighbouring peaks were merged while some other peaks were missing. Thus, the usage of Mylar film hindered identification of the Posaconazole polymorphic form (Figure 5).

In the XRPD pattern of the precipitate covered with the transparent pressure-sensitive tape, peaks of high intensity at 14.1, 16.9, 18.6 and 25.6 2-theta appeared because of the tape while the peaks of the precipitate were barely visible (Figure 5). As a result, the pressure-sensitive tape cannot be applied as a protective material for detection of the Posaconazole polymorphic form.

The transparent food membrane was the only material which had no interference with the peaks of the precipitate. The XRPD pattern of the sample that was protected with the transparent food membrane was practically identical to the respective pattern recorded without the use of the membrane, i.e., Posaconazole remained as Form-S (Figure 5).

### 2.6. Stability of Posaconazole API in the Oral Suspension′s Precipitate Covered with Transparent Food Membrane

**Figure 6:**
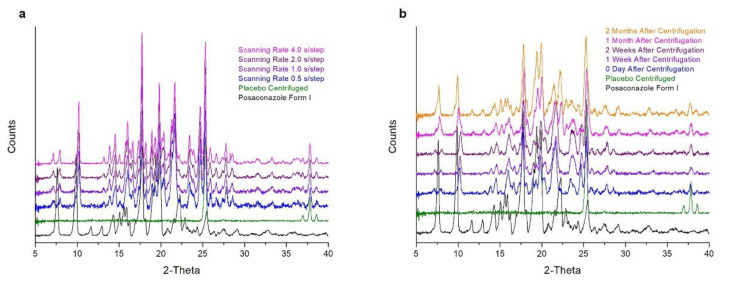
Stabilization of Posaconazole Form-S in the oral suspension′s precipitate after covering the sample with transparent food membrane: (**a**) the XRPD patterns of Posaconazole oral suspension′s precipitate recorded with different scanning rates at 0.5 s/step (blue line), 1.0 s/step (violet line), 2.0 s/step (purple line) and 4.0 s/step (magenta line); of the precipitate of Posaconazole oral suspension′s placebo (green line); and of Posaconazole Form I (black line) and (**b**) the XRPD patterns of Posaconazole oral suspension′s precipitate immediately after centrifugation and enfolded with the transparent food membrane (blue line) after 1 week (violet line), 2 weeks (purple line), 1 month (magenta line) and 2 months (orange line); of the precipitate of Posaconazole oral suspension′s placebo (green line); and of Posaconazole Form I (black line).

The ability of the food membrane to protect the sample from polymorphic conversion was tested not only under ambient conditions but also during XRPD recording. The precipitate was covered with the membrane, and the pattern was recorded with gradually increasing scanning rates from 0.5 s/step to 1.0 s/step, 2.0 s/step and finally 4.0 s/step. No difference was observed among the patterns recorded with the four different scanning rates. Posaconazole in all four patterns remained as Form-S. When a slower scanning speed (2.0 s/step or 4.0 s/step) was used, a pattern with higher signal/noise ratio was obtained (Figure 6a).

The stability of Posaconazole Form-S in the precipitate when covered with the transparent food membrane was studied also against time. It was found that the membrane delayed the polymorphic conversion from Posaconazole Form-S to Form I for 2 weeks. After 1 month, both Posaconazole Form-S and Form I were identified in the precipitate by XRPD. However, after 2 months, Posaconazole was converted completely to Form I (Figure 6b). With the use of the membrane, a very significant delay in the polymorphic conversion of Posaconazole Form-S to Form I was achieved compared to the 1 h required for conversion without the membrane. Therefore, the transparent food membrane was efficient in trapping water molecules in the precipitate, delaying Posaconazole polymorphic conversion and giving the opportunity for more time-consuming analysis in order to obtain an XRPD pattern of higher quality.

## 3. Discussion

**Figure 7:**
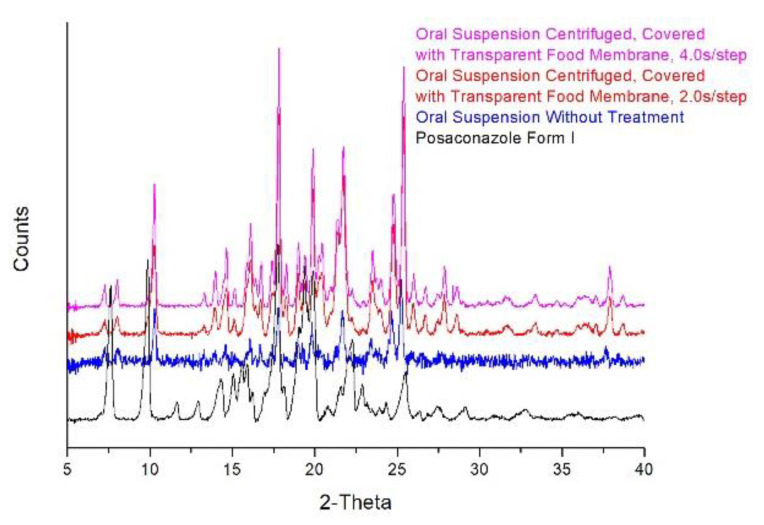
Comparison of Posaconazole oral suspension without treatment and Posaconazole oral suspension treated with the developed method: XRPD patterns of Posaconazole Form I (**black line**), Posaconazole oral suspension without treatment (**blue line**) and Posaconazole oral suspension centrifuged and covered with the transparent food membrane recorded with 2.0 s/step (**red line**) and 4.0 s/step scanning rate (**magenta line**).

Posaconazole oral suspension treated with the developed sample-preparation method resulted in an XRPD pattern that was practically identical to the untreated Posaconazole oral suspension. Comparison of the patterns of the precipitate recorded with 2.0 s/step or 4.0 s/step against the initial untreated oral suspension revealed that the signal/noise ratio increased significantly (Figure 7). This improvement in the quality of Posaconazole oral suspension′s XRPD pattern gave the opportunity for complete characterization of Posaconazole Form-S and excluded the possibility of impurity from any other polymorphic form of Posaconazole. Hence, using the developed method, the S/N ratio of the XRPD pattern of the suspension increased and the S polymorph was stabilized. This method is fast, reliable, environmentally friendly and capable of identifying the crystal form of the API in oral suspensions.

In this study, the characteristic 2-theta and the respective d-spacing of the XRPD pattern′s peaks of Posaconazole Form-S were determined and compared to those corresponding to Posaconazole Form I (Table 1). The characteristic peaks of Posaconazole Form-S are at 7.2, 7.9, 10.2, 13.2, 13.9, 14.5, 15.0, 16.0, 16.6, 17.3, 17.7, 18.1, 18.9, 19.3, 19.8, 20.2, 20.9, 21.3, 21.6, 22.1, 23.4, 23.7, 24.7, 25.9, 26.6, 27.4, 27.7, 28.5, 30.3, 31.0, 31.6, 33.2, 34.5, 35.2, 35.8, 36.5, 36.8, 37.4 and 38.5 2-theta. The existence of those peaks and the absence of the peaks at 7.6, 9.8, 11.1, 11.6, 12.9, 14.3, 15.6, 15.8, 16.2, 17.1, 19.1, 22.9, 23.1, 23.9, 24.3, 25.5, 26.3, 26.9, 29.2, 32.3, 32.8, 33.5, 35.5, 36.0, 37.9, 38.2 and 39.5 2-theta peaks can be used in order to differentiate Posaconazole Form-S from Posaconazole Form I in the oral suspension.

Previous attempts to identify the polymorphism of the API in the reference commercial Posaconazole oral suspension, Noxafil^®^, were reported [20]. However, the non-controlled treatment of suspension (drying and centrifugation) resulted in XRPD patterns that matched the pattern of Form I [20]. Also, in the scientific discussion of Noxafil^®^ by the European Medicines Agency [19], it is reported that Posaconazole is produced as Form I and in the whole manufacturing process, even if the final oral suspension remains in the same crystal form [19].

**Figure 8:**
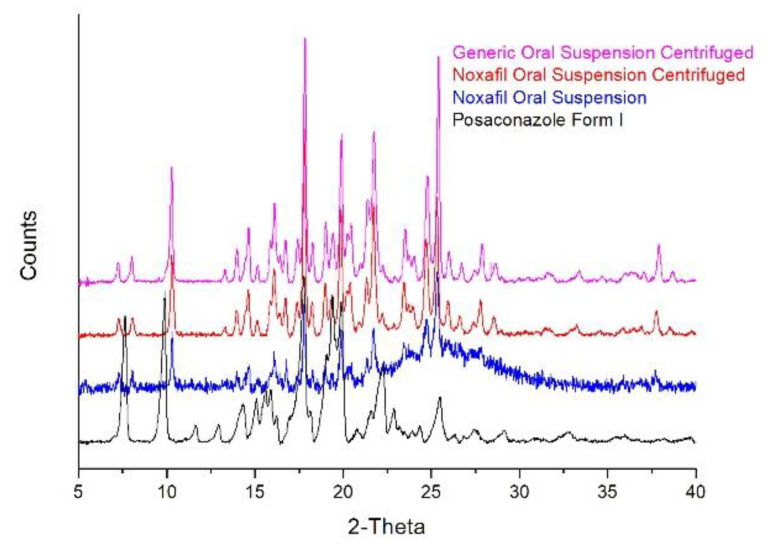
Comparison of the generic Posaconazole oral suspension and the reference Posaconazole oral suspension, Noxafil^®^, treated with the developed sample-preparation method: The XRPD patterns of Posaconazole Form I (**black line**), Noxafil^®^ Posaconazole oral suspension (**blue line**), Noxafil^®^ Posaconazole oral suspension centrifuged and covered with the transparent food membrane (**red line**) and generic Posaconazole oral suspension centrifuged and covered with the transparent food membrane (**magenta line**).

This study is the first to report that the Posaconazole API in oral suspensions is Form-S. The reference Noxafil^®^ oral suspension was also analysed using XRPD, and the developed sample-preparation method was applied. It was found that, after centrifugation of Noxafil^®^, the XRPD pattern of its precipitate was practically identical to the pattern of the precipitate of the generic Posaconazole oral suspension (Figure 8). Moreover, the XRPD pattern of the Noxafil^®^ precipitate highly resembled the pattern of the untreated Noxafil^®^ oral suspension (Figure 8) while Posaconazole Form I was not detected in the suspension.

The developed sample-preparation method is likely to be also efficient in the identification of the crystal form of the API in other suspensions and formulations containing water molecules in which the concentration of the API is rather low, like oral suspensions (other than Posaconazole oral suspension and otic suspensions) and ophthalmic suspensions. Moreover, the use of the proposed method could be extended to identification of the crystal form of the API in semi-solid dispersions, such as o/w creams. Furthermore, application of the transparent food membrane for covering the sample would be useful for determining the polymorphic form of the API in environmentally sensitive samples, which are susceptible to polymorphic conversion.

## 4. Materials and Methods

### 4.1. Materials and Samples

Posaconazole API Form I; Posaconazole oral suspensions (40 mg/mL); and Posaconazole oral suspension′s placebo, composed of Polysorbate 80, sodium citrate monohydrate, citric acid monohydrate, simethicone, xanthan gum, sodium benzoate, liquid glucose, glycerin, artificial cherry flavour, titanium dioxide and purified water, were kindly provided by the Greek pharmaceutical company GENEPHARM S.A (Pallini, Attica, Greece). The commercial reference Posaconazole oral suspensions, Noxafil^®^, was acquired from a local drug store.

### 4.2. X-Ray Powder Diffraction (XRPD)

For identification of the polymorphism of the Posaconazole API in oral suspensions, an X-ray Powder Diffractometer (Bruker AXS D2 Phaser 2nd Gen, Karlsruhe, Germany) was used, equipped with a standard Bragg Brentano geometry with fixed primary and linear LYNXEYE (1D mode) detector. Ceramic X-ray tube KFL Cu-2K, 0.4 mm × 12 mm, with a Ka spectral line (λ = 1.54184 Å) was used as the incident radiation. The tube worked with 300 W (30 kV voltage and 10 mA current). The scan mode was continuous, and a locked coupled scan type was used. The step size was 0.02° (2θ), the scan speed varied while the region of 2–40° (2θ) was scanned. No rotation was applied for recording the XRPD patterns. A 0.6-mm primary divergence slit, a 3-mm air scatter screen, an 8-mm anti-scatter slit, a 2.5° soler slit and a 5° (2θ) opening for the Position Sensitive Detector (PSD), were used. Polymethylmethacrylate (PMMA) XRPD sample holders with a 25-mm diameter and a 0.5-mm-deep circular cavity for spreading the sample were used. The background of the XRPD patterns was subtracted using the software DIFFRAC.SUITE EVA V4.1.1.

#### 4.2.1. Posaconazole Oral Suspension Analysis via XRPD

The Posaconazole API was loaded on the PMMA sample holder by simply spreading the powder using a 25 mm × 75 mm × 1 mm glass slide. The XRPD pattern of Posaconazole Form I was recorded using a scan rate of 0.2 s/step. All other settings were the same as those previously mentioned.

Regarding Posaconazole oral suspension, reference Posaconazole oral suspension, Noxafil^®^ and the suspension′s placebo, the sample was shaken evenly and placed on the PMMA sample holder with a Pasteur pipette, avoiding bubbles in the samples. Subsequently, their XRPD patterns were recorded with a scan speed of 1.0 s/step.

#### 4.2.2. Sample-Preparation Methods for Increasing Posaconazole′s Concentration in Oral Suspensions

In order to overcome the problem of a low signal/noise ratio in the XRPD pattern of the oral suspension, three different methods were tested to increase the concentration of the API in the oral suspensions by separating the solid components of the suspension from the liquid excipients.

The first method tested was filtration under vacuum: 5 mL of Posaconazole oral suspension (40 mg/mL) were filtered using 0.22-μm GSWP nitrocellulose membrane filters (Merck Millipore Ltd., Cork, Ireland) or borosilicate glass microfibre filter circles MN GF-1 with a retention capacity of 0.7 μm (Macherey-Nagel, Düren, Germany) or cellulose filter circles MN 619 de with a retention capacity of 1–2 μm (Macherey-Nagel, Düren, Germany) on a Büchner funnel or on a crucible funnel and a vacuum pump (KNF Neuberger Inc. Laboport, Trenton, NJ, USA). As the d(4.3) of Posaconazole API was found at 7.42 μm and the d(4.3) of the oral suspension was 6.72 μm (Hydro SV detection unit, Mastersizer 3000, Malvern Panalytical, Malvern, UK), filters with higher retention capacity were not used. The oral suspension could not be filtered in any case, and no XRPD pattern was obtained.

The second method was drying of the oral suspension at room temperature: 5 mL (5.4 g) of Posaconazole oral suspension (40 mg/mL, approximately 3.7% *w*/*w*) was placed on a borosilicate glass Petri dish (70 mm diameter × 15 mm height) after shaking the suspension′s bottle evenly, and the suspension was left to dry overnight. The received solid was sticky and gummy, and its weight was found at approximately 1.6 g. Subsequently, the XRPD pattern of the dried oral suspension was recorded using a scan rate of 0.5 s/step.

The third method was centrifugation of the oral suspension. Different rpms (2000 rpm, 4000 rpm, 6000 rpm and 8000 rpm), temperature (10 °C, 15 °C, 20 °C and 25 °C), times (13 min, 23 min and 33 min) of centrifugation and amount of sample (2.5 mL, 5 mL, 10 mL and 15 mL) were tested and validated before selecting the most appropriate conditions. Among the different rpms, 8000 rpm was selected since no precipitate formed when 2000 rpm and 4000 rpm were used while, at 6000 rpm, the precipitate was not completely recovered. The temperature of 25 °C was found to be the most appropriate, as solid components were found in the supernatant when lower temperatures were used. When the suspension was centrifuged for 13 min, the precipitate was not fully recovered as solid components could be detected in the supernatant. The centrifugations of 23 min and 33 min resulted in practically identical XRPD patterns with no expenses in the recovery ratio. Thus, 23-min centrifugation was selected as the aim was to develop a fast sample-preparation method. Concerning the amount of sample, 5 mL was selected, as all amounts resulted in the same recovery ratio and our aim was to develop a method with the smallest amount possible; however, the PMMA sample holder of the XRPD requires approximately 1 mL to be loaded and this amount of precipitate is recovered when at least 5 mL is centrifuged.

Hence, 5 mL (5.4 g) of Posaconazole oral suspension (40 mg/mL, approximately 3.7% *w*/*w*) was placed in a 50-mL falcon tube and the sample was centrifuged at 8000 rpm and 25 °C for 23 min using a refrigerated centrifuge (Heraeus Biofuge Stratos, Kendro, Osterode, Germany). The supernatant, which was approximately 4 mL (4.4 g), was removed, and the precipitate was collected. The precipitate was loaded on a PMMA sample holder using a spatula, and the XRPD pattern was recorded using a scan rate of 0.5 s/step. For evaluation of the API′s recovery from the Posaconazole oral suspension, artificial Posaconazole Form I water dispersions were prepared in triplicates, they were centrifuged and then left to dry at ambient temperature overnight. Each dispersion was prepared by adding 200 mg of Posaconazole Form I in purified water (15 MΩ·cm at 25 °C, Elix, Merck Millipore, Darmstadt, Germany), and the ratio of the API recovered after centrifugation and drying of the dispersion were determined (Table 2). Full recovery was achieved. Moreover, the ratio of precipitate recovered after centrifugation of the oral suspension was determined and it was found equal to 19.22% ± 0.77% (Table 3). For validating the precision and reproducibility of centrifugation, the oral suspension was centrifuged and analysed in triplicates every 4 h in the same day, and for validating the repeatability and ruggedness of the process, it was done in triplicates for three consecutive days. In addition, this method was applied to three different batches of the oral suspension. 

#### 4.2.3. Setting the Scanning Rate of the XRPD Analysis

The separated precipitate after centrifugation was loaded on a PMMA sample holder, and its XRPD pattern was recorded using four different scanning rates; 0.5 s/step, 1.0 s/step, 2.0 s/step and 4.0 s/step scan speeds were applied. For each XRPD pattern, a fresh precipitate was prepared.

#### 4.2.4. Studying the Stability of Posaconazole in Oral Suspension′s Precipitate

For determination of the time effect on Posaconazole′s polymorphism in the oral suspension′s precipitate isolated by centrifugation, its XRPD pattern was recorded immediately after centrifugation (0 min) and after 30 min, 60 min, 90 min and 120 min using a scanning rate of 0.5 s/step.

#### 4.2.5. Sample-Preparation Method for Delaying the Polymorphic Conversion

Different types of covers were used to avoid water loss from the precipitate. For each type of cover, a fresh suspension was centrifuged and the respective precipitate was collected.

A commercially available airtight specimen sample holder with dome-like x-ray transparent cap for environmentally sensitive materials (Bruker, Karlsruhe, Germany) was tested. The precipitate was placed on the airtight specimen sample holder, and the dome-like cap was screwed on the sample holder. The XRPD pattern of the sample was recorded using a 1.0 s/step scanning rate, and no air scatter screen was used. All other settings were the same.

The precipitate was transferred on a PMMA sample holder and covered airtight with a 75 mm × 50 mm biaxially oriented polyethylene terephthalate (PET) film: Mylar film (DuPont Teijin Films, Luxembourg S.A.). The XRPD pattern of the precipitate covered with Mylar film was acquired with a 1.0 s/step scanning speed.

The precipitate was spread on a PMMA sample holder with a spatula, and it was covered with three pieces (40 mm length each) of transparent pressure-sensitive tape (Scotch tape, 12 mm width, 3M, Greece). The XRPD pattern of the precipitate covered with transparent pressure-sensitive tape was recorded using 1.0 s/step scanning rate.

A piece of 120 mm × 130 mm and 0.006-mm-thick polyethylene low-density transparent food membrane (Vileda Freshmate 50 m, Aspropyrgos, Greece) was used to enfold the precipitate loaded on a PMMA sample holder. The XRPD pattern of the sample covered with the transparent food membrane was obtained with a 1.0 s/step scanning rate.

#### 4.2.6. Study of Stability of the Posaconazole API in the Oral Suspension′s Precipitate Covered with a Transparent Food Membrane

The Posaconazole oral suspension was centrifuged, and the precipitate was separated, loaded on a PMMA sample holder and covered diligently with a transparent food membrane in order to prevent water loss. The XRPD pattern of the sample was acquired using four different scanning rates: 0.5 s/step, 1.0 s/step, 2.0 s/step and 4.0 s/step.

The same procedure was followed for preparing the sample in order to study the stability of Posaconazole in the precipitate when using the transparent food membrane. The XRPD pattern of the sample was recorded immediately after centrifugation (0 days) and after 1 week, 2 weeks, 1 month and 2 months. Each time, a scanning rate of 0.5 s/step was used.

### 4.3. Spectral Analysis

The software OriginPro 8 (OriginLab Corporation, Northampton, MA, USA) was used for creation of the graphs and for analysis of the XRPD patterns.

## 5. Conclusions

To conclude, a sample-preparation method was developed for characterisation of the crystal form of the Posaconazole API in oral suspensions using XRPD. More specifically, the API can be isolated as a precipitate together with the other undissolved excipients from the oral suspensions using centrifugation. However, the Posaconazole API is prone to water loss and is instable, and a polymorphic conversion to the thermodynamically stable Form I is rapid. In order to overcome the stability issue, a transparent food membrane was used to cover the precipitate loaded on the PMMA sample holder and a slower scanning rate of 2.0 s/step or 4.0 s/step was applied to increase the signal/noise ratio. Application of this novel sample-preparation method in Posaconazole oral suspensions revealed that the crystal form of Posaconazole in oral suspensions is Form-S.

## Figures and Tables

**Table 1:** The characteristic 2-theta diffraction angles and d-spacing of the XRPD patterns of Posaconazole Form I and Posaconazole Form-S.

Posaconazole Form I		Posaconazole Form-S	
2-Theta Angles (Degrees) ^1^	d-Spacing (Å)	2-Theta Angles (Degrees) ^1^	d-Spacing (Å)
7.6	11.63	7.2	12.35
9.8	8.98	7.9	11.17
11.1	7.94	10.2	8.69
11.6	7.61	13.2	6.70
12.9	6.85	13.9	6.39
14.3	6.20	14.5	6.10
15.1	5.88	15.0	5.89
15.6	5.68	16.0	5.54
15.8	5.59	16.6	5.33
16.2	5.47	17.3	5.11
17.1	5.19	17.7	5.01
17.7	5.00	18.1	4.89
18.1	4.90	18.9	4.69
19.1	4.64	19.3	4.60
19.4	4.57	19.8	4.49
19.9	4.47	20.2	4.39
20.8	4.26	20.9	4.25
21.6	4.11	21.3	4.16
22.2	4.00	21.6	4.10
22.9	3.89	22.1	4.01
23.1	3.84	23.4	3.80
23.5	3.79	23.7	3.75
23.9	3.71	24.7	3.61
24.3	3.66	25.9	3.44
25.5	3.50	26.6	3.35
26.3	3.39	27.4	3.26
26.9	3.31	27.7	3.21
27.5	3.24	28.5	3.13
29.2	3.06	30.3	2.95
31.0	2.88	31.0	2.88
32.3	2.77	31.6	2.83
32.8	2.73	33.2	2.69
33.5	2.67	34.5	2.59
35.5	2.53	35.2	2.55
36.0	2.50	35.8	2.51
36.7	2.45	36.5	2.46
37.9	2.37	36.8	2.44
38.2	2.36	37.4	2.40
39.5	2.28	38.5	2.34

^1^ The 2-theta diffraction angles may vary by ±0.2° [21].

**Table 2:** Mass of water dispersion, separated supernatant, recovered precipitate and recovered dried precipitate as well as the recovery ratio of the Posaconazole API after centrifugation of Posaconazole water dispersion in triplicates.

	Sample Mass (g)	Supernatant Mass (g)	Precipitate Mass (g)	Dried Precipitate Mass (g)	Recovery Ratio From the Initial API Mass ^1^ (%)
Sample 1	4.964	4.210	0.751	0.196	97.90
Sample 2	5.023	4.226	0.798	0.200	100.05
Sample 3	4.988	4.258	0.728	0.202	100.80
Average	4.992	4.231	0.759	0.199	99.58
Standard Deviation	0.030	0.024	0.035	0.003	1.51

^1^ The initial Posaconazole active pharmaceutical ingredient (API) mass in all three samples was 0.200 g.

**Table 3:** Mass of oral suspension, separated supernatant and recovered precipitate as well as the recovery ratio of the precipitate after centrifugation of the Posaconazole oral suspension in triplicates.

	Sample Mass (g)	Supernatant Mass (g)	Precipitate Mass (g)	Recovery Ratio ^1^ (%)
Sample 1	5.454	4.425	1.029	18.87
Sample 2	5.250	4.190	1.056	20.11
Sample 3	5.413	4.399	1.012	18.70
Average	5.372	4.338	1.032	19.22
Standard Deviation	0.108	0.129	0.022	0.77

^1^ The recovery ratio was calculated as a 100% ratio of the precipitate mass divided by the mass of the respective sample.
